# Effects of the Duration of Zilpaterol Hydrochloride Supplementation and Days on Feed on Performance, Carcass Traits and Saleable Meat Yield of Nellore Bulls

**DOI:** 10.3390/ani11082450

**Published:** 2021-08-20

**Authors:** Mariana Caetano, Rodrigo S. Goulart, Saulo L. Silva, Paulo R. Leme, Sérgio B. Pflanzer, Antonio C. R. dos Santos, Dante P. D. Lanna

**Affiliations:** 1 Davies Livestock Research Centre, Department of Animal and Veterinary Bioscience, School of Animal and Veterinary Sciences, Roseworthy Campus, The University of Adelaide, Roseworthy, SA 5371, Australia; mariana.caetano@adelaide.edu.au; 2 Department of Animal Science, College of Animal Science and Food Engineering, University of São Paulo, Pirassununga 13635-900, SP, Brazil; sauloluz@usp.br (S.L.S.); prleme@usp.br (P.R.L.); 3 Department of Food Technology, Faculty of Food Engineering, The University of Campinas, Campinas 13083-862, SP, Brazil; pflanzer@fea.unicamp.br; 4 Department of Animal Science, College of Agriculture, University of São Paulo, Luiz de Queiroz, Piracicaba 13418-900, SP, Brazil; ar755@cornell.edu (A.C.R.d.S.); dplanna@usp.br (D.P.D.L.)

**Keywords:** beef cattle, beta-adrenergic receptor agonist, *Bos indicus*, feedlot, Nellore

## Abstract

**Simple Summary:**

Zilpaterol hydrochloride (ZH) is a *β*-adrenergic agonist (*β*AA) to be feed to feedlot cattle at a rate of 8.3 mg/kg during the final 20 to 40 d of the finishing period followed by a minimum 3 d withdrawal period. This compound has the potential to increase animal performance, improve carcass weight and meat yield. Although significant information regarding the effects of duration of ZH supplementation and days on the feed of *Bos taurus* cattle has been provided, there is a lack of information relative to its effects on *Bos indicus* breeds such as Nellore cattle. The current study aimed to evaluate the effects of the duration of ZH supplementation and DOF on performance, carcass characteristics, and saleable meat yield of Nellore bulls. The HCW and total saleable meat yield linearly increased with the duration of ZH supplementation as well as when the length of the feedlot period increased. We recommend supplementing ZH for Nellore bulls at least for 20 days, independently of days on feed, to improve hot carcass weight, hindquarter, and saleable meat yields of Nellore bulls.

**Abstract:**

This study evaluated the effects of the duration of ZH supplementation and days on feed (DOF) on performance, carcass characteristics, and saleable meat yield of Nellore young bulls. The fixed effects included the duration (0, 20, 30, or 40 d before slaughter plus a 3 d ZH withdrawal period—8.33 mg of ZH/kg of DM) and DOF (90 and 117 d). Feed efficiency (G:F) linearly increased when the duration of ZH supplementation increased (*p* < 0.01). Nellore bulls fed ZH had greater HCW (*p* < 0.01), dressing percentage (*p* < 0.01) and Longissimus muscle area (LMA) (*p* < 0.01), but less 12th-rib fat (*p* = 0.04) than the control group. The hot carcass weight (HCW) (*p* < 0.01), and dressing percentage increased linearly (*p* < 0.01) with the increase of duration of ZH supplementation. The HCW, ossification, and 12th-rib fat increased with DOF (*p* < 0.01). The ZH supplemented group had most of the individual cuts of hindquarters and total saleable meat increased compared with the control. Zilpaterol hydrochloride was effective in improving hot carcass weight, hindquarter, and saleable meat yields of Nellore bulls when fed for at least 20 d before slaughter, independently of days on feed.

## 1. Introduction

Over the years, the length of the feedlot period in Brazil has been increased substantially, on average from 80 to 100 days on feed (DOF) [1,2] and some producers and feedlot technicians have mentioned that the DOF may continue increasing in the future motivated by better prices for finished cattle, aiming for ideal live weights or better premiums offered by meatpackers [3]. In contrast, advancing DOF will promote a reduction in beef cattle performance fed high-grain-based diets due to the increase in energy required per unit of gain as cattle approach a mature BW [4]. To overcome some of these disadvantages of cattle fed in feedlots, mainly in the last days, the use of *β*AA has been investigated in finishing diets. Zilpaterol hydrochloride (ZH), a *β*AA, has the potential to increase weight gain, improve feed efficiency, and increase carcass leanness [5,6,7,8]. The ZH supplementation in feedlots, during the last 20 to 40 DOF, can offset high feed costs and increase profitability in cattle finished in feedlots. However, the magnitude of enhancement, the efficiency of gain, modification of carcass characteristics, and meat quality of beef cattle are influenced by the duration of treatment with *β*AA [9].

Previous studies evaluated the effects of duration of ZH supplementation and days on the finishing diet on feedlot cattle [5,10], however, most research investigated the effects of ZH in *Bos taurus* breeds. Since *Bos indicus* breeds are produced in tropical areas around the world and significantly contribute to the red meat industry, further information about the effects of ZH supplementation in *Bos indicus* cattle is needed. The objectives of the current study were to evaluate the effects of the duration of ZH supplementation and DOF on performance, carcass characteristics, and saleable meat yield of Nellore bulls.

## 2. Materials and Methods

### 2.1. Animal, Experimental Design and Treatments

Ninety-six Nellore bulls (377 ± 25 kg initial BW and 24 months of age) were used in a randomized complete block design with a 4 × 2 factorial arrangement of treatments to test interactions between duration of ZH supplementation (0, 20, 30, or 40 d followed by a 3-d withdrawal period before slaughter; 8.33 mg/kg; Zilmax^®^, MSD Animal Health, São Paulo, SP, Brazil) and DOF before slaughter (90 and 117 d). Bulls were blocked by initial BW and randomly assigned, within a block, to treatments and individual gates and pens. All animals were individually fed using individual pens and Calan gates. Forty-eight bulls were housed in four pens (10 × 16 m; 12 bulls/pen; 14.5 m^2^/bull) equipped with electronic gates (American Calan Inc., Northwood, NH, USA) that allowed individual control of feed intake. The other forty-eight bulls were housed in individual pens (6 × 3 m; 18 m^2^/bull). Both types of pens had concrete floors and partial roof coverings. Bulls had free access to fresh water during the entire study period.

### 2.2. Management and Diets

Before entry to the feedlot, cattle received an s.c. injection of clostridial vaccine (Sintoxan Polivalente T, Merial Brazil, Campinas, SP, Brazil), ivermectin for control of internal and external parasites (1 mL/50 kg of BW; Ourofino Agronegócio, Cravinhos, SP, Brazil), and vitamin A, D and E (Ourofino Agronegócio, Cravinhos, SP, Brazil).

Bulls were fed twice daily, with half of the diet offered at 0700 h and the remainder at 1600 h. Diets were mixed using a total feed mixer (Casale Unimix 1200, Casale Equipamentos, São Carlos, SP, Brazil), and then weighed into nylon bags using a fixed scale (Toledo do Brazil, Indústria de Balanças Ltda, 2124/1, São Bernardo do Campo, SP, Brazil) to provide *ad libitum* intake. To avoid cross-contamination, the diet without ZH was always mixed first, followed by the ZH diet. The feed mixer was cleaned after each mixture and washed by the end of the day. Once ZH feeding started, the feeding order was established and was based on the duration of ZH feeding, with the 40-d group fed first, followed by the 30, and 20 d duration groups.

Diets were formulated to meet or exceed the beef cattle recommendations for nutrients [4] (Table 1). The ingredients and chemical composition of the diets used are given in Table 1. Daily feed allocations were based on bunk scores (Pritchard, 1998). Refusals were removed and weighed twice weekly or when necessary due to rainfall.

### 2.3. Feed Samples

Feed subsamples were collected weekly during the experiment and dried at 55 °C for 24 h to adjust diet composition based on DM basis. Diets were also subsampled on a weekly basis and stored at −20 °C until the end of the experiment when they were bulked on a proportionate basis to create a composite sample. Each composite sample was dried in a forced-air oven at 55 °C for 72 h, and ground in a Willey mill using a 1 mm screen (MA-680 Marconi Ltda, Piracicaba, SP, Brazil). Subsamples were analyzed in duplicates for the DM at 105 °C (methods 934.01) [12], crude protein (CP; method 954.01) [13]; ether extract (method 920.29) [12], neutral detergent fiber (nonsequential and ash-free) [14], and acid detergent fiber (method 973.18) [13].

### 2.4. Performance, Blood Parameters and Carcass Traits

Bodyweight was measured fortnightly and at the beginning and the end of the trial. In addition, BW was also collected prior to and post to the ZH treatment. The average daily gain was calculated by linear regression of unfasted BW over time [15]. Blood samples (1 × Serum, 2 × EDTA plasma tubes; 10 mL each) were collected on d 43 (as a baseline), and 3 prior to slaughter via jugular venipuncture. Whole blood was collected in the EDTA, and serum tubes were centrifuged (2420× *g*/10 min) and 1.5 mL aliquots of either serum or plasma were frozen in Eppendorf tubes. Blood serum parameters were analyzed using commercial colorimetric assay kits for urea, glucose (Laborlab, Guarulhos, SP, Brazil), and NEFA (Randox Laboratories, São Paulo, SP, Brazil) in a Novaspec Plus spectrophotometer (Amersham Biosciences, Uppsala, Sweden).

After 90 d (*n* = 48) and 117 d (*n* = 48), bulls were slaughtered in a commercial abattoir (Lins, São Paulo, Brazil) following the Sanitary and Industrial Inspection Regulation for Animal Origin Products [16]. Hot carcass weight (HCW) and dressing percentage were determined immediately after evisceration. Renal, pelvic, and inguinal fats were collected and weighed from the left half of each carcass, and values were expressed as % of HCW. Twenty-four h after slaughter, digital images of the *Longissimus* muscle area (LMA) were obtained between the 12th and 13th ribs. The LMA and backfat thickness (BFT) images were evaluated using Lince^®^ software (M&S Consultoria Agropecuaria, Pirassununga, SP, Brazil). The marbling score was evaluated according to the Meat Evaluation Handbook: Beef Grading [17].

### 2.5. Saleable Meat Yield

**Figure 1:**
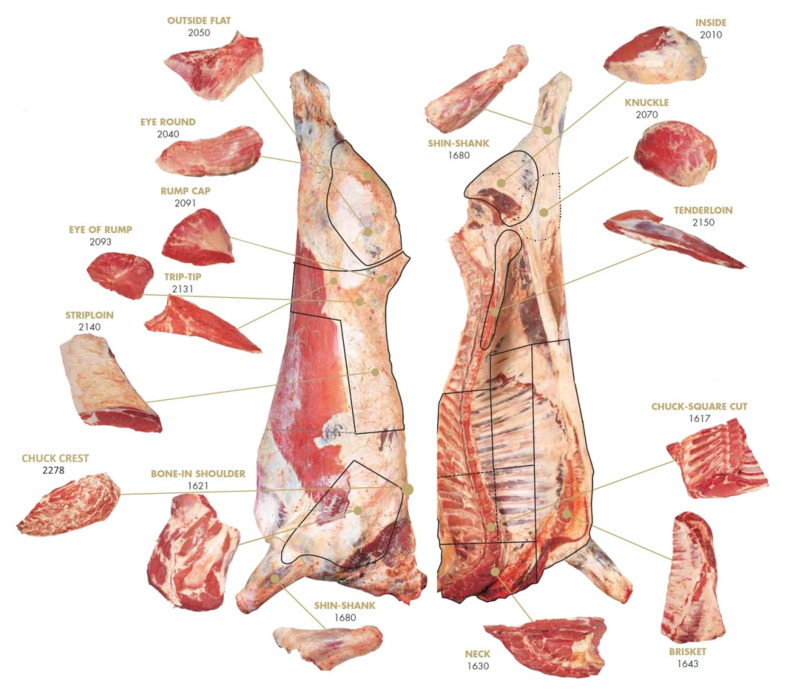
Forequarter and hindquarter boneless cuts evaluated to determine the saleable meat yield (Adapted from UNECE Standards-Bovine Meat Carcasses and Cuts [18]).

Forty-eight hours post-mortem, carcasses were boned and total saleable meat, primal forequarter, and hindquarter cuts [18] from the left carcass side of all animals were weighed (Figure 1). The evaluated cuts were shoulder (# 1621), shank fore (# 1680), neck and chuck (# 1630 + 1617), brisket (# 1643), chuck crest (# 2278), striploin (# 2140), tenderloin (# 2150), top sirloin cap (# 2091), trip-tip (# 2131), eye of rump (# 2093), inside (# 2010), outside flat (# 2050), eye of round (# 2040), knuckle (# 2070), and shank hind (# 1680). The saleable meat yield of subprimal cuts was expressed as the percentage of the cold carcass weight.

### 2.6. Statistical Analysis

Plots of residuals and the W statistic [19] were evaluated to determine normality for all data, and outliers (>3 and <−3 standard deviation) were excluded. Animal performance, carcass traits at slaughter, and saleable meat yield were analyzed using mixed model procedures (Proc Mixed; SAS Inst. Inc., Cary, NC, USA). The model included the duration of ZH supplementation (0, 20, 30, or 40 d before slaughter), DOF (90 and 117 d) and the interaction between the duration of ZH and DOF as fixed effects, block (initial BW) as a random effect, and the animal was the experimental unit. The blood and ultrasound carcass traits were evaluated as repeated measurements, considering the same fixed effects with a further component including the day of measurement and their two and three-way interactions. The covariance structure of residuals was modeled and the best fit for every trait was used.

For significant effects of treatments, pairwise comparisons were made among least-square means using the PDIFF option. In addition, orthogonal contrasts were used to compare: (1) Control vs. ZH supplemented groups; and (2) linear and quadratic effects of duration of ZH administration. Effects were considered significant when *p* ≤ 0.05.

## 3. Results

### 3.1. Performance

There was no interaction between the duration of ZH supplementation and DOF for performance characteristics (Table 2). The initial BW was similar among treatments at the start of the trial. There was no effect on the duration of ZH supplementation for the final BW, ADG, and DMI. Increases in the duration of ZH supplementation resulted in linear increases in G:F (*p* < 0.01). The final BW was 4.5% greater (*p* < 0.01) when cattle were fed for 117 d instead of 90 d. As expected, bulls fed finishing diets for 117 d decreased ADG (*p* < 0.01) and G:F (*p* < 0.01) by 10% compared to those bulls finished at 90 d.

### 3.2. Carcass Characteristics

No significant interaction between the duration of ZH supplementation and DOF was observed for any of the carcass characteristics (Table 3). The LMA was greater for bulls finished for 117 d and supplemented with ZH for 30 to 40 d before slaughter. The HCW of bulls fed ZH were heavier (*p* < 0.01) compared to those fed a control diet. The ZH supplementation increased dressing percentage (*p* < 0.01), and reduced BFT (*p* = 0.04) and renal, pelvic, and inguinal fat (*p* < 0.01) compared to those animals not supplemented. The HCW (*p* < 0.01) and dressing percentage (*p* < 0.01), linearly increased with the duration of ZH supplementation. Renal, pelvic, and inguinal fat was quadratically associated with the duration of ZH supplementation (*p* = 0.01; Table 3) with a reduction from 0d to 20d with a similar value among 20, 30, and 40 d groups. The marbling score was reduced by 22% when bulls were fed ZH compared to the control group (146.6 vs. 188.0; *p* = 0.03). There was a trend of a linear reduction in the marbling score as the duration of ZH supplementation increased (*p* = 0.06). Ossification was not affected by the duration of ZH supplementation. When bulls were fed for 117 d, HCW, ossification, and carcass fats (BFT and renal, pelvic, and inguinal fat) were greater than those slaughtered at d 90 (*p* < 0.01). There was no effect of DOF on dressing percentage, LMA, and marbling score.

### 3.3. Blood Parameters

There was an interaction between DOF and blood sampling for blood glucose and serum urea nitrogen (*p* < 0.01; Table 4). When bulls were confined for 90 d, blood glucose was greater prior than post zilpaterol supplementation (80.69 vs. 66.10 mg/dL). However, no difference in blood glucose was observed before and after zilpaterol supplementation for bulls confined for 117 d (63.09 vs. 65.94 mg/dL). On the other hand, serum urea nitrogen was higher prior to zilpaterol supplementation for bulls confined for 117 d (18.10 vs. 15.47 mg/dL). However, there was no difference in serum urea nitrogen prior and post zilpaterol supplementation for bulls confined for 90 d (15.76 vs. 16.57 mg/dL). Lower blood serum urea nitrogen was observed for bulls fed ZH in comparison to the non-supplemented group (16.17 vs. 17.54 mg/dL; *p* = 0.03). A quadratic association of the duration of ZH supplementation was observed for blood serum urea nitrogen (*p* < 0.01) with the lowest values of serum urea observed when bulls were fed zilpaterol for 20 and 30 d (15.50 and 16.00 mg/dL, respectively) and the highest values obtained for the control group and bulls fed zilpaterol for 40 d before slaughter (17.60 and 16.82 mg/dL, respectively). Blood NEFA was higher in bulls fed for 117d compared to those fed for 90 d in the feedlot (0.116 vs. 0.137 mmol/L; *p* = 0.03). There was also an effect of blood sampling for NEFA, with the highest values of blood NEFA observed post zilpaterol supplementation (0.117 vs. 0.137 mmol/L; *p* = 0.03). However, blood NEFA and glucose were not affected by ZH.

### 3.4. Saleable Meat Yield

There was no interaction between DOF and the duration of ZH for single cuts and total saleable meat yields (Table 5). Total saleable meat yield linearly increased as the duration of ZH supplementation increased (*p* < 0.01). The Nellore bulls fed ZH had greater yields of tenderloin (*p* = 0.02), eye of rump (*p* < 0.01), inside (*p* < 0.01), outside flat (*p* < 0.01), eye of round (*p* < 0.01), knuckle (*p* < 0.01), shank hind (*p* < 0.01) in comparison to a control (without ZH). Tenderloin (*p* < 0.01), inside (*p* < 0.01), and outside flat (*p* < 0.01) yields increased linearly when the duration of ZH supplementation increased. A quadratic effect of the duration of ZH was observed for the eye of round (*p* = 0.01), and knuckle (*p* < 0.01) yields, with values increasing from 0 d to 20 d and then being similar among ZH supplemented groups. There was no significant effect of the duration of ZH supplementation in the shoulder, shank fore, neck and chuck, brisket, chuck crest, striploin, top sirloin cap, shank hind, and trip-tip yields. There was a trend of a linear increase of shoulder yield when the duration of ZH supplementation increased (*p* < 0.07). Total saleable meat yield increased when DOF increased from 90 d to 117 d (*p* < 0.01). Shoulder, neck and chuck, brisket, and chuck crest yields increased when animals were fed 27 days longer (*p* < 0.01). However, bulls fed longer (117 d) decreased shank fore, tenderloin, inside, the eye of round, knuckle yields (*p* < 0.01), and top sirloin cap (*p* = 0.03). There was no significant effect of DOF in striploin, trip-tip, the eye of rump, outside flat, and shank hind.

## 4. Discussion

### 4.1. Performance

Previous studies have shown an improvement in G:F, HCW, dressing percentage, total retail cuts, and yield of saleable cuts when Nellore cattle were fed ZH for 20 [6,20] and 30 d before slaughter [7,21,22]. However, there is still a lack of information focusing on Nellore cattle at the minimum and maximum treatment duration, during the last 20 to 40 d plus 3 d withdraw before slaughter across DOF. According to the literature, all available studies evaluating the effects of duration of ZH supplementation and days on the finishing diet on feedlot cattle were conducted with *Bos taurus* breeds [5,10]. Knowing that there are significant differences in the nutritional efficiency, growth rates, composition of gain (differences in muscle and adipose tissue deposition), and carcass characteristics between different genetic groups of beef cattle [4,23,24], studies focusing on Nellore cattle over DOF and duration of ZH supplementation are necessary.

In the present study, no difference in final BW was observed between the control and ZH treatments. Similar to our results, other trials did not find a statistical difference in final BW between control and ZH supplementation [5,21,25]. Several studies have reported that ZH improved G:F in feedlot cattle due to the negative effect of ZH supplementation on DMI or a positive effect of this *β*AA on ADG [5,7,21,25,26]. Montgomery et al. [27] conducted experiments at 3 US locations (CA, ID, and TX) to determine the effects of the duration of ZH feeding of finishing steers and heifers and reported that when ZH was fed for 20 to 40 d before slaughter, the feed intake of animals decreased. On the other hand, Elam et al. [28] investigated the effect of ZH on DMI over the entire feeding period in four experiments conducted at commercial US feedlots, and these authors did not observe a significant change in DMI when cattle were fed ZH for 20, 30, or 40 d before slaughter. In addition, it has been suggested that no adjustment to DMI should be performed when ZH was added in finishing diets [4]. In the current study, there was no effect of the duration of ZH feeding on DMI during the entire trial, but cattle fed ZH in the last 20 to 40 d in the feedlot improved G:F by 8.77% when compared to the control treatment.

In the current trial, when DOF increased to 117 d, final BW increased by 4.5%, and ADG and G:F decreased by 10.4% and 9.4%, respectively; however, no difference was observed on DMI. Our results are in agreement with those found by Vasconcelos et al. [5], who did not find differences for DMI (8.73 to 8.88 kg/d) when DOF was increased from 136 to 198 d; however, these authors noted that the final BW increased, and the feed efficiencies decreased as time on feed increased. In the same way, Van Koevering et al. [29] mentioned that increasing DOF for 105, 119, 133, and 147 d, resulted in a linear increase of the final BW of British × Continental crossbred steers (472, 499, 518, and 529 kg of BW), but no difference was observed in feed intake. It is expected that animals synthesize more fat towards the end of the finishing period, which requires more energy per gram of tissue than muscle. Therefore, when the DOF was increased from 90 to 117 d, the ADG and G:F was negatively impacted in the current study.

### 4.2. Carcass Traits

Zilpaterol hydrochloride supplementation for 40 d increased HCW by 18 kg, while those supplemented for 20 or 30 d increased HCW by 13 kg compared to the control group. Thus, 40 d ZH supplementation promoted the greatest HCW and consequently was the most effective treatment for Nellore bulls finished on high-grain-based diets. Vasconcelos et al. [5] reported that there were no appreciable additive gains in carcass measurements of British and British x Continental steers when the duration of feeding ZH was beyond 20 d. On the other hand, Strydom and Smith [25] demonstrated that Bonsmara cows had better growth performance and trimmed meat yield when 30 d ZH supplementation was adopted rather than 20 and 40 d ZH supplementation.

The use of *β*AA, such as ZH, in feedlot cattle can lead to the same empty body fat (as a percentage of empty BW) at a greater BW, due to the effect of increased protein synthesis and decreased protein degradation. This effect suggests that ZH can increase the mature body size of cattle compared to the control group [4,30]. These results are consistent with the findings of Leheska et al. [31], who noted that the percentage of empty body fat was smaller for steers fed ZH, resulting from decreased BFT and quality grade, and increased HCW and LMA. In the current study, Nellore bulls not supplemented ZH had greater marbling scores, BFT, and renal, pelvic, and inguinal fat than cattle fed diets containing ZH (20, 30, and 40 d before slaughter). Additionally, our data showed that there was a trend to linearly decrease marbling score as the duration of ZH supplementation increased.

Several studies have shown that increased DOF was associated with increased hot carcass weight and BFT reports [29,32,33], which is in line with our results where the HCW, BFT and renal, pelvic, and inguinal fat increased substantially as DOF increased. However, increases in HCW and BFT were not associated with an increase in dressing percentage in our study. Van Koevering et al. [29] also did not observe differences in dressing percentage of British and Continental crossbred yearling steers fed for 105, 119, 133, and 147 d. However, Vasconcelos et al. [5] and Owens and Gardner [32] observed a linear increase of dressing percentage as days on feed increased. In spite of discrepant results regarding the effect of DOF on dressing percentage, it can be suggested that the impact of DOF on dressing percentage is highly dependent on cattle fed for longer durations (196 or 270 d) and carcass fatness [29,34].

It is worthwhile to mention that according to Owens and Gardner [32] dressing percentage typically has been associated with increases in carcass weight and fat deposition, however, dressing percentage is more correlated with LMA than with BFT. This can partially explain the lack of difference found in this work for dressing percentage (and LMA), in spite of a greater BFT for animals fed longer (117 d), compared to the 90 d fed group. Complementary to this, the dressing percentage is also affected by the digestive tract, including fat and lean, and its content, and in this study, animals fed for 117 d had greater renal, pelvic, and inguinal fat which contributed to the reduction of a dressing percentage of this group.

### 4.3. Blood Parameters

Previous studies reported a decrease of serum urea in animals fed ZH, due to either an increase in tissue nitrogen deposition or a decrease of protein catabolism in muscle tissue [29,35,36,37]. As expected, in the present study, serum urea was 8.5% greater in the control group compared to the ZH group. However, the diet was sufficient to supply an adequate level of amino acids for greater synthesis of muscle when bulls were fed ZH. The concentration of NEFA was not affected by ZH supplementation in the current study. The concentration of NEFA has been used as an indicator of lipolysis since fat stores are mobilized when there is a greater energy demand for physiological functions. The concentration of NEFA in the serum of animals fed ZH has been inconsistent. Parr et al. [36] observed an increase in serum NEFA concentrations in response to the use of ZH. However, several studies, including the current study, did not find any effect of ZH in serum NEFA concentrations [29,37,38]. On the other hand, Cônsolo et al. [6] observed that serum NEFA and glucose concentrations were lower in ZH fed than that in the control group. In the current study, there was no difference in blood glucose concentration for bulls fed ZH in comparison with the control group. The blood glucose concentration was very similar across all treatments 3 d prior to slaughter. However, blood glucose concentration was lower when bulls were confined for 117 d due to higher energetic demand to synthesize fat tissue towards the end of the current trial [39]. Greater blood NEFA concentration observed in bulls confined for 117 d represents a different metabolic stage in comparison to those bulls confined for 90 d [40].

### 4.4. Saleable Meat Yield

In the current study, ZH supplemented animals had a higher total saleable meat yield and higher yields for the majority of the cuts from the hindquarter than the non-supplemented group. Overall, 7 of the 11 cuts from the hindquarter were all significantly higher in bulls supplemented with ZH for at least 20 d when compared to the non-supplemented group. In general, cuts from the leg (eye of rump, inside, outside flat, eye of round, knuckle, and shank hind) were highly impacted by ZH supplementation, compared to the loin, where only tenderloin yield was increased in ZH supplemented animals. On the other hand, no impact on saleable meat yield from the forequarter was observed between ZH supplemented and non-supplemented animals.

The results obtained in the current trial evaluating the effect of ZH on saleable meat yield in *Bos indicus* breeds, such as Nellore, are in agreement with previous studies. Cônsolo et al. [20] evaluated the effect of ZH feeding in the last 30 d out of 118 d of the finishing period and found that ZH increased the hindquarter by 13% when measured as total weight, and this *β*AA increased bone yield in Nellore heifers. Brigida et al. [22] reported heavier weights of saleable cuts in Nellore bulls and immunocastrated cattle receiving ZH in the last 30 d, with no difference in saleable meat in the forequarter. Likewise, Costa et al. [7] showed that the addition of ZH in the diet 20 d prior to slaughter increased yield of subprimal commercial cuts in both Nellore bulls and steers. The ZH has consistently demonstrated the ability to increase muscle deposition in Nellore cattle regardless of gender. Of particular interest, this increase has occurred in the hindquarter muscles, which are the most valuable carcass cuts and can substantially impact industry profitability. In addition, it is important to elucidate that, knowing that the modern livestock industry aims to produce more food using fewer inputs as competition for land, water, and energy intensifies, Capper and Hayes [41] mentioned that the use of growth-enhancing technologies, such as *β*-adrenergic agonist and other compounds, would increase both the economic and environmental sustainability of the industry.

Increasing DOF from 90 to 117 days resulted in higher yields for the majority of cuts from the forequarter, however, the opposite was observed in the hindquarter, where animals fed for 90 d showed higher yields in 7 out 10 cuts evaluated. It is well-known that the forequarter proportion increases as the intact males became older [42,43,44], and consequently, the yield of cuts of forequarter also increases, which can explain why bulls fed longer (117 d) had mostly, higher cuts yields than those fed 90 d. In addition, it is worthwhile to point out that testosterone has an increased effect on the growth of the forequarter and neck muscles of males, and at the age of the animals used in this study (24 months old), testosterone has an active role on animal metabolism. In contrast, the same fact can explain the higher yields of hindquarter cuts observed in animals fed 90 days, once there is an increase in forequarter proportion there will be a proportional decrease in hindquarter proportions and cuts yields, as observed in this work.

## 5. Conclusions

Zilpaterol hydrochloride was effective in improving hot carcass weight, hindquarter, and saleable meat yields of Nellore bulls when fed for at least 20 d before slaughter, independently of days on feed. Therefore, the ZH compound could be a valuable tool to increase meat production in feedlot systems under tropical conditions. Continuous investigation into the possible impacts of ZH on the meat quality of these types of animals and feeding conditions seem to be important to validate this technology.

## Figures and Tables

**Table 1:** Composition of the experimental diets (DM basis).

	Adaptation 1 ^1^	Adaptation 2 ^2^	Control Finishing Diet	ZH Finishing Diet
*Ingredient, % of DM*				
Corn silage	50.00	34.00	15.00	15.00
Finely ground dry corn	26.00	48.00	69.66	69.66
Cottonseed hulls	15.00	10.78	10.00	10.00
Soybean meal	6.00	4.00	2.00	2.00
Urea	0.70	1.00	1.20	1.20
Limestone	1.58	1.48	1.04	1.04
Potassium chloride	-	-	0.38	0.38
Sodium chloride	0.10	0.12	0.10	0.10
Dicalcium phosphate	0.10	0.10	0.10	0.10
Mineral premix ^3^	0.52	0.52	0.52	-
Mineral premix + ZH ^3,4^	-	-	-	0.52
*Chemical composition, %* ^5^				
DM	54.50	60.88	72.06	72.10
TDN	76.07	79.09	82.86	82.86
Crude protein	14.44	14.43	14.49	14.30
Ether extract	4.56	4.14	5.58	5.14
NDF	34.86	27.18	22.09	21.79
ADF	20.50	17.19	11.23	11.21

^1^ Diet was offered to the first 6 d of adaptation; ^2^ Diet was offered from 7 until 12 d; ^3^ Mineral premix contained, per kilogram, 45.4 g Ca, 73.4 g S, 34.9 g P, 30.9 g Mg, 213.1 g Na, 82 mg Co, 1.871 mg Cu, 1.000 mg Fe, 130 mg I, 3.670 mg Mn, 26 mg Se, 5.564 mg Zn, 290.000 IU vitamin A, and 2.533 mg salinomycin (equivalent to 13 mg/kg of diet DM); ^4^ The zilpaterol hydrochloride (ZH) premix supplied ZH at 8.33 mg/kg (DM basis); ^5^ Dry matter (DM), Neutral detergent fiber (NDF), Acid detergent fiber (ADF), Total digestible nutrients (TDN) calculated according to [11].

**Table 2:** Least square means, standard error of the mean (SEM), and probabilities of performance, according to the duration of zilpaterol hydrochloride (ZH) supplementation and days on feed (DOF).

	DOF, d		SEM	*p*-Value	Duration of ZH Supplementation, d ^1^					*p*-Value		
Item ^2^	90	117			0	20	30	40	SEM	0 vs. ZH ^3^	L ^3^	Q ^3^
*Performance*												
Initial BW, kg	376	376	10.8	0.93	378	377	377	372	11.1	0.52	0.28	0.58
Final BW, kg	555	580	9.4	<0.01	561	571	566	571	10.6	0.31	0.43	0.69
ADG, kg	1.93	1.73	0.043	<0.01	1.75	1.86	1.81	1.91	0.06	0.11	0.12	0.83
DMI, kg/d	10.1	9.9	0.22	0.39	10.2	10.1	9.9	9.8	0.27	0.32	0.18	0.94
G:F	0.192	0.174	0.005	<0.01	0.171	0.184	0.182	0.194	0.006	<0.01	<0.01	0.86

^1^ Treatment diets were formulated to provide no ZH (0 d) or ZH (8.33 mg/kg, DM basis) for the last 20, 30, or 40 d on the finishing period for 90 or 117 d on the finishing diet before slaughter; ^2^ BW—body weight; ADG—average daily gain; DMI—dry matter intake; G:F—gain to feed ratio; ^3^ 0 vs. ZH—Control vs. ZH supplemented groups; Linear (L) and quadratic (Q) effects of duration of ZH administration.

**Table 3:** Least square means, standard error of the mean (SEM), and probabilities of carcass traits according to the duration of zilpaterol hydrochloride (ZH) supplementation and days on feed (DOF).

	DOF, d		SEM	*p*-Value	Duration of ZH Supplementation, d ^1^					*p*-Value		
Item	90	117			0	20	30	40	SEM	0 vs. ZH ^2^	L ^2^	Q ^2^
Hot carcass weight, kg	311	323	6.30	<0.01	306	319	319	324	7.10	<0.01	<0.01	0.37
Dressing, %	56.0	55.8	0.23	0.51	54.6	55.9	56.3	56.7	0.30	<0.01	<0.01	0.10
Ossification ^3^	61.0	76.2	3.72	<0.01	67.1	67.1	65.0	75.4	5.24	0.76	0.33	0.32
Marbling Score ^4^	154	159	12.0	0.76	188	154	148	138	17.0	0.03	0.06	0.50
*Longissimus* muscle area, cm^2^	73.4	74.1	1.83	0.64	68.0	73.2	76.8	76.9	2.14	<0.01	<0.01	0.11
Backfat thickness, mm	4.27	5.42	0.19	<0.01	5.38	4.63	4.72	4.67	0.27	0.04	0.10	0.20
Renal, pelvic, and inguinal fat, %	3.50	4.20	0.11	<0.01	4.35	3.64	3.69	3.66	0.14	<0.01	<0.01	0.01

^1^ Treatment diets were formulated to provide no ZH (0 d) or ZH (8.33 mg/kg, DM basis) for the last 20, 30, or 40 d on the finishing period for 90 or 117 d on the finishing diet before slaughter; ^2^ 0 vs. ZH—Control vs. ZH supplemented groups; Linear (L) and quadratic (Q) effects of duration of ZH administration; ^3^ Ossification—measure of physiological maturity of the beef carcass. 0 to 99—A maturity; 100 to 199—B maturity; >200—C maturity; ^4^ Marbling score. Practically devoid—100; Traces—200.

**Table 4:** Least square means, standard error of the mean (SEM) and probabilities of blood parameters, according to the duration of zilpaterol hydrochloride (ZH) supplementation and days on feed (DOF).

	DOF, d		SEM	*p*-Value	Duration of ZH Supplementation, d ^1^					*p*-Value		
Item	90	117			0	20	30	40	SEM	0 vs. ZH ^3^	L ^3^	Q ^3^
*Blood parameters*												
Glucose, mg/dL	73.39	64.52	2.85	<0.01	71.15	66.23	68.08	70.35	3.12	0.25	0.95	0.05
NEFA ^2^, mmol/L	0.116	0.137	0.012	0.03	0.134	0.112	0.129	0.131	0.013	0.33	0.84	0.20
Urea nitrogen, mg/dL	16.17	16.79	0.44	0.25	17.60	15.50	16.00	16.82	0.57	0.03	0.43	<0.01

^1^ Treatment diets were formulated to provide no ZH (0 d) or ZH (8.33 mg/kg, DM basis) for the last 20, 30, or 40 d on the finishing period for 90 or 117 d on the finishing diet before slaughter; ^2^ NEFA—non-sterified fatty acids; ^3^ 0 vs. ZH—Control vs. ZH supplemented groups; Linear (L) and quadratic (Q) effects of duration of ZH administration.

**Table 5:** Least square means, standard error of the mean (SEM) and probabilities of subprimal and total saleable meat yield, according to the duration of zilpaterol hydrochloride (ZH) supplementation and days on feed (DOF).

	DOF, d		SEM	*p*-Value	Duration of ZH Supplementation, d ^1^					*p*-Value		
Item	90	117			0	20	30	40	SEM	0 vs. ZH ^2^	Linear ^2^	Q ^2^
Forequarter	40.65	40.60	0.195	0.86	40.89	40.58	40.52	40.53	0.276	0.28	0.32	0.67
Shoulder, %	8.61	8.82	0.053	<0.01	8.58	8.72	8.73	8.82	0.074	0.07	0.06	0.78
Shank fore, %	2.82	2.63	0.038	<0.01	2.77	2.77	2.72	2.66	0.048	0.36	0.06	0.42
Neck and Chuck, %	13.7	14.2	0.150	<0.01	14.1	14.2	13.9	13.8	0.200	0.53	0.17	0.73
Brisket, %	5.34	5.68	0.076	<0.01	5.61	5.42	5.53	5.48	0.108	0.31	0.59	0.50
Chuck crest, %	3.92	4.45	0.124	<0.01	4.12	4.16	4.09	4.37	0.175	0.69	0.38	0.51
Hindquarter	46.4	45.5	0.192	<0.01	45.02	46.26	46.26	46.35	0.0271	<0.01	<0.01	0.11
Striploin, %	6.89	6.91	0.063	0.74	6.83	6.88	7.02	6.86	0.083	0.36	0.50	0.17
Tenderloin, %	2.18	2.09	0.022	<0.01	2.08	2.11	2.18	2.19	0.029	0.02	<0.01	0.79
Top sirloin cap, %	1.62	1.54	0.032	0.03	1.57	1.58	1.63	1.55	0.041	0.75	0.93	0.27
Trip-tip, %	1.18	1.21	0.017	0.28	1.17	1.18	1.20	1.23	0.024	0.23	0.08	0.74
Eye of rump, %	3.26	3.20	0.037	0.28	3.09	3.25	3.25	3.30	0.052	<0.01	<0.01	0.52
Inside, %	7.39	7.17	0.055	<0.01	6.95	7.20	7.40	7.56	0.077	<0.01	<0.01	0.53
Outside flat, %	4.40	4.45	0.040	0.31	4.26	4.41	4.41	4.56	0.056	<0.01	<0.01	0.62
Eye of round, %	2.05	1.97	0.023	<0.01	1.91	2.05	2.05	2.03	0.030	<0.01	<0.01	<0.01
Knuckle, %	4.39	4.20	0.051	<0.01	4.11	4.35	4.39	4.35	0.061	<0.01	<0.01	<0.01
Shank hind, %	3.31	3.25	0.027	0.86	3.18	3.32	3.31	3.30	0.038	<0.01	0.06	0.06
Saleable meat yield, %	74.6	75.1	0.170	<0.01	73.9	75.0	75.0	75.4	0.230	<0.01	<0.01	0.15

^1^ Treatment diets were formulated to provide no ZH (0 d) or ZH (8.33 mg/kg, DM basis) for the last 20, 30, or 40 d on the finishing period for 90 or 117 d on the finishing diet before slaughter; ^2^ 0 vs. ZH—Control vs. ZH supplemented groups; Linear (L) and quadratic (Q) effects of duration of ZH administration.
